# Tracking of body adiposity indicators from childhood to adolescence: Mediation by BMI

**DOI:** 10.1371/journal.pone.0191908

**Published:** 2018-02-06

**Authors:** Enio R. V. Ronque, André O. Werneck, Maria R. O. Bueno, Edilson S. Cyrino, Luiz C. R. Stanganelli, Miguel Arruda

**Affiliations:** 1 Study and Research Group in Physical Activity and Exercise–GEPAFE. State University of Londrina–UEL, Londrina, Brazil; 2 Study and Research Group in Metabolism, Nutrition, and Exercise–GEPEMENE. State University of Londrina–UEL, Londrina, Brazil; 3 Center of Physical Education and Sport, State University of Londrina, Londrina, Brazil; 4 Faculty of Physical Education, State University of Campinas, São Paulo, Brazil; GeneDx, UNITED STATES

## Abstract

Our aim was to verify the tracking of body adiposity indicators from childhood to adolescence and analyze the mediation effects of BMI on the stability of body adiposity. Our sample was composed by 375 children (197 boys). The children were followed-up over 3 years. Body mass and stature were measured as anthropometric indicators. Body adiposity was estimated through the subcutaneous skinfold method, with measures of triceps (TRSF) and subscapular skinfolds (SSSF). Skinfolds were analyzed singly and agglutinated through the sum of skinfolds (∑SF). The sample was categorized into tertiles, and thereafter, the kappa coefficient and McNemar test were adopted to verify stability. For continuous measures, the Intra-Class Correlation coefficient (ICC) was used. Moreover, mediation analyzes were used according to Baron and Kenny with the Sobel test to verify mediation effects. The significance level adopted was 5%. Adiposity indicators increased during the 3 years of follow-up in both sexes (p<0.05). ICCs in all indicators of adiposity were between 0.84 and 0.94 for boys and 0.86 and 0.94 for girls, indicating high tracking. Moreover, 70% of subjects remained in the highest tertile of body adiposity. However, no differences were observed in tertile changes (p>0.05). BMI at the age of adiposity rebound partially mediated all indicators of adiposity from childhood (baseline) to adolescence (3 years later) in both sexes (p<0.001). Thus, moderate to high tracking of body adiposity indicators between childhood and adolescence was verified. Moreover, BMI at the age of adiposity rebound partially mediated the relationship between adiposity in childhood (baseline) and in adolescence (3 years of follow-up).

## Introduction

An excess of body fat in children and adolescents is associated with the development of several cardiovascular risk factors, such as hypertension, type 2 diabetes, hypercholesterolemia, and others [1–3]. Moreover, excess body adiposity during childhood and adolescence also increases the risk of cardiovascular risk factors and metabolic syndrome in older ages (adulthood) [4], contributing to the increase in mortality rates [5,6]. Furthermore, an excess of body fat during childhood and adolescence can track to adulthood; more than a half obese adolescents become obese adults.

Given this context, obesity during childhood and adolescence has become a public health priority [7] and interventions aiming at the normalization of the ponderal index of body adiposity should be conducted during early ages to minimize morbidity/mortality risk in adulthood [5,7]. In this way, the central aspect of studies of behaviors and characteristics of subjects during childhood and adolescence and posteriorly adulthood, refers to the concept of tracking, which is the tendency of remain in the same channel or relative position of a given attribute within the group over time [8,9]. As the transition between childhood and adolescence is a period of several changes in body dimensions as body mass, stature, and adiposity [10–12], verifying the kinetic of body adiposity during this transition should be an important strategy in combating obesity.

Moreover, there are three critical periods for the development of childhood and adolescent obesity [13]: the prenatal period, which refers to early development during gestation and is commonly represented by birth weight; the adiposity rebound, which begins at the moment the body mass index (BMI) increases after the lowest point during infancy, which can occur at after 7 years old [14,15]; and biological maturation, which is related to development of adiposity during adolescence [13,16].

Adiposity rebound has been criticized for the lack of physiological mechanisms and a possible statistical origin of this marker [17], but posteriorly studies have shown that even subjects that present a low BMI during childhood, suffering an early rebound, they are more likely to present higher obesity and consequently, cardiovascular risk during adulthood, than their peers [14,15]. Another issue is the real effect of adiposity during the adiposity rebound period or even closely to the rebound, given that other tissues also growth during these years and stature has an increase peak after adiposity rebound [18]. In this sense, it is not clear what tissue most affect adiposity rebound and what tissue most growth with adiposity rebound, having in mind that adiposity rebound can also have a complex interaction with other behavioral and biological variables [19,20]. In this way, our aim was to verify the tracking of body adiposity from childhood to adolescence and analyze the mediation of BMI in the maintenance of body adiposity during three years of follow-up.

## Materials and methods

### Sample and design

Data were extracted from a prospective study named “Analysis of growth and health-related physical fitness in schoolchildren of high socioeconomic status”. The design of the study was longitudinal mixed with four birth years of cohorts (1992, 1993, 1994, and 1995), with ages between 7 and 10 years old at baseline (2002, 2003, 2004, and 2005), participants were followed-up over 3 years. Data were collected from a school in the center of the city to attend the criteria of sampling (α = 95%, statistical power = 80%, and error of 5%). The final sample of this study was 375 subjects (197 boys). Inclusion criteria were being enrolled in the school, having the established chronological age, having an interest in participating in the study, and presenting the consent term signed by the subject and child’s parents. Subjects with incomplete data were excluded. All procedures were approved by the local ethics committee, according to the declaration of Helsinki (Ethics committee of UNICAMP’s medical sciences faculty, process: 249/2002). More information about the design of the study and sampling have been published previously [21].

### Anthropometry

Body mass was obtained through a digital scale with a precision of 0.05 kg. Stature was acquired through a stadiometer with a 0.1 cm precision, according to standardized procedures. From body mass and stature measures, BMI was estimated (body mass (kg) / stature (m)^2^).

To estimate body adiposity, triceps and subscapular skinfolds were collected using a Lange caliper (*Cambridge Scientific Industries*, *Inc*., *Cambridge*, *Maryland*), according to procedures described by Harrison et al. [22]. Based on the measures of triceps and subscapular skinfolds, results were interpreted individually (by skinfold) and through the sum of skinfolds. To verify the tracking, the sample was divided into tertiles by indicator of body adiposity according to sex and chronological age, using percentiles as cutoffs (33.3 and 66.6: ≤ P° 33.3 tertile 1; > 33.3 but ≤ P° 66.6 tertile 2; > P° 66.6 tertile 3).

### Statistical procedures

To characterize the sample, we used mean and standard deviation values, and the t test for repeated measures with values of effect size (%Δ). Tracking was analyzed through the application of three statistical procedures: a) Intra class correlation coefficients (ICC) were used for continuous data of obesity indicators with observation of Confidence Intervals (CI 95%). Values lower than 0.30 were considered low, between 0.30 and 0.60 moderate, and > 0.60 high [9]; b) the McNemar test was used for the variance between tertiles; and c) To verify agreement (tracking) between proportions of subjects the Kappa coefficient (k) was used, k < 0.20 was considered low; k between 0.41 and 0.60, moderate; k between 0.61 and 0.80, high and k > 0.80, very high [23].

The mediation analyzes were performed according to the principles of Baron and Kenny [24]. In the first instance, the mediator variable (BMI at the age of adiposity rebound) was regressed onto the independent variable (subscapular skinfold, triceps skinfold and sum of skinfolds at childhood). In the second equation, the dependent variable (subscapular skinfold, triceps skinfold and sum of skinfolds at adolescence) was regressed onto the independent variable. Finally, in the third equation, the dependent variable was regressed onto the independent variable, adjusted for the mediator. Mediation was identified if the following criteria were met: (a) in the first equation, the independent variable was a significant predictor of the mediator; (b) in the second equation, the independent variable was a significant predictor of the dependent variable; (c) in the third equation, the mediator was a significant predictor of the dependent variable and the association between the dependent and independent variables (from equation two) was either partially or fully removed [24]. The data were analyzed using SPSS software version 22.0 (SPSS Inc., Chicago, Illinois, USA), with a significance level of 5%.

## Results

Characteristics of the sample are presented in Table 1, with means and standard deviations according to sex at baseline and after 3 years of follow-up. Values of anthropometric measures as well as body adiposity increased in both sexes (p < 0.05).

**Table 1:** Characteristics of the sample, presented as means and standard deviation, with comparisons between baseline and after 3 years of follow-up.

	Boys (n = 197)				
	Baseline	3 years follow-up	*T*	*p*	*MD (Δ%)*
Chronological age (years)	8.9 ± 1.1	11.9 ± 1.1	457.05	< 0.001	3.0 (33.7)
Body mass (kg)	33.5 ± 8.8	47.7 ±12.7	37.14	< 0.001	14.2 (42.3)
Stature (cm)	134.6 ± 8.5	152.9 ± 10.5	74.89	< 0.001	18.3 (13.6)
BMI (kg/m^2^)	18.2 ± 3.3	20.1 ± 3.8	15.66	< 0.001	1.9 (10.4)
TRSF (mm)	13.7 ± 5.9	18.5 ± 7.7	13.11	< 0.001	4.8 (35.0)
SSSF (mm)	9.7 ± 6.3	14.9 ± 9.0	12.58	< 0.001	5.2 (53.6)
∑SF (mm)	23.4 ± 11.8	33.4 ± 16.3	13.61	< 0.001	10.0 (42.7)
	Girls (n = 178)				
	Baseline	3 years follow-up	*T*	*p*	*MD (Δ%)*
Chronological age (years)	9.0 ± 1.1	12.0 ± 1.1	535.00	< 0.001	3.0 (33.3)
Body mass (kg)	32.0 ± 8.2	46.1 ± 11.9	35.37	< 0.001	14.1 (44.0)
Stature (cm)	133.9 ± 8.9	152.6 ± 9.7	82.72	< 0.001	18.7 (13.9)
BMI (kg/m^2^)	17.6 ± 3.0	19.5 ± 3.6	15.78	< 0.001	1.9 (10.8)
TRSF (mm)	15.1 ± 5.2	19.9 ± 6.9	15.18	< 0.001	4.8 (31.8)
SSSF (mm)	10.8 ± 6.2	14.3 ± 7.5	9.82	< 0.001	3.5 (32.4)
∑SF (mm)	25.9 ± 11.0	34.2 ± 13.9	13.49	< 0.001	8.3 (32.0)

BMI, body mass index; TRSF, triceps skinfold; SSSF, subscapular skinfold; ∑SF, sum of skinfolds (TRSF + SSSF); MD, mean difference; Δ% = effect size [(mean 3 years follow-up—mean baseline) / mean baseline) * 100].

In the case of anthropometric measures, we highlight that body mass increased more than 40% for boys (33.5 vs. 47.7; p < 0.001) and girls (32.0 vs. 46.1; p < 0.001), while stature and BMI increased by only 14% and 10% respectively. For body adiposity, greater accumulation of subcutaneous fat in boys was noted, according to the ∑SF (43% vs. 32%), probably caused by the increase in adiposity in the trunk region (9.7 vs. 14.9; p < 0.001) in relation to 32% observed in girls (10.8 vs. 14.3; p < 0.001).

Tracking of continuous adiposity indicators are presented in Table 2. All adiposity indicators presented high tracking from childhood to adolescence, with ICCs varying between 0.84 and 0.94 (p<0.001). In Table 3, the percentage of subjects that remained in the same tertile or changed to a lower or upper tertile of body adiposity between childhood and adolescence can be observed, according to sex. Among boys, 64%, 71%, and 69% of subjects remained in the same tertile for TRSF, SSSF, and ∑SF respectively. For girls, 63% for TRSF, 60% for SSSF, and 62% for ∑SF remained in the same tertile. Although the McNemar test was not statistically significant, change rates among boys were between 13% and 18% and for girls around 20%.

**Table 2:** Intra-class correlation coefficient (ICC) and confidence intervals (CI 95%) of body adiposity indicators between baseline and after 3 years of follow-up according to sex.

Body adiposity		*ICC*	*CI 95%*	*p*	*Classification*
Boys (n = 197)	BMI	0.94	0.92–0.96	< 0.01	High
	TRSF	0.84	0.79–0.88	< 0.01	High
	SSSF	0.84	0.78–0.88	< 0.01	High
	∑SF	0.85	0.80–0.89	< 0.01	High
Girls (n = 178)	BMI	0.94	0.91–0.95	< 0.01	High
	TRSF	0.87	0.82–0.90	< 0.01	High
	SSSF	0.86	0.81–0.90	< 0.01	High
	∑SF	0.88	0.84–0.91	< 0.01	High
Total (n = 375)	BMI	0.94	0.92–0.95	< 0.01	High
	TRSF	0.85	0.82–0.88	< 0.01	High
	SSSF	0.84	0.81–0.87	< 0.01	High
	∑SF	0.86	0.83–0.89	< 0.01	High

BMI, body mass index; TRSF, triceps skinfold; SSSF, subscapular skinfold; ∑SF, sum of skinfolds (TRSF + SSSF).

**Table 3:** *Tracking* (%) of subjects that maintained in the same tertile or changed to a lower tertile (LT) or higher tertile (HT) of body adiposity indicators according to sex.

Adiposity	*% Tracking*	LT—%	HT—%	*Kappa*
	Boys (n = 197)			
TRSF	64.0	18.3	17.7	0.45*
SSSF	70.6	13.2	16.2	0.55*
∑SF	69.1	16.2	14.7	0.53*
	Girls (n = 178)			
TRSF	62.9	18.6	18.5	0.44*
SSSF	60.0	19.7	20.3	0.40*
∑SF	61.8	19.7	18.5	0.42*

TRSF, triceps skinfold; SSSF, subscapular skinfold; ∑SF, sum of skinfolds (TRSF + SSSF).

* P < 0.001.

**Fig 1:**
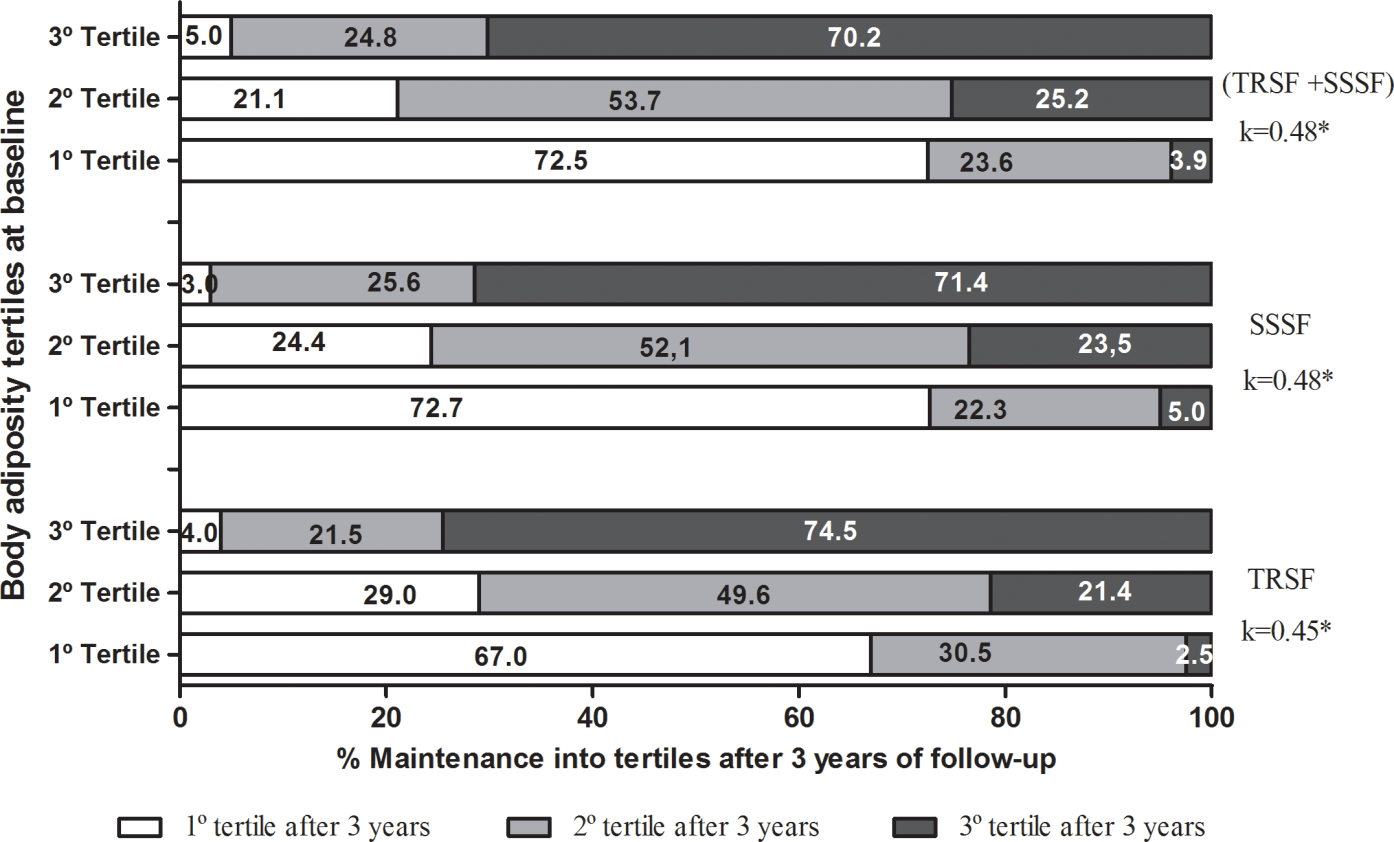
Proportion of subjects that maintained or changed their tertiles of body adiposity during 3 years of follow-up. Note: TRSF, triceps skinfold; SSSF, subscapular skinfold; ∑SF, sum of skinfolds (TRSF + SSSF). * P < 0.001.

When analyzing regardless of sex, it was noted that 74.5%, 71.4%, and 70.2% of subjects that were in the highest tertile in childhood, remained in the same tertile in adolescence for TRSF, SSSF, and ∑SF respectively. Similarly, 67%, 72.7%, and 72.5% of subjects that were in the lowest tertile at baseline, remained in the lowest tertile after 3 years. Concerning the 2^nd^ tertile, about half the subjects remained in the same tertile after 3 years for TRSF, SSSF, and ∑SF (Fig 1).

On the other hand, 24%, 28.5%, and 29.1% of subjects changed from the lowest and medium tertiles (1 and 2) to the highest tertile (3), respectively for TRSF, SSSF, and ∑SF, while only 4%, 3%, and 5% changed from the highest tertile to the lowest tertile, for TRSF, SSSF, and ∑SF, respectively. In addition, for all indicators of body adiposity, regardless of sex, the McNemar test did not identify differences (p > 0.05).

Maintenance of the total tracking (Fig 1) for adiposity indicators during 3 years of follow-up was considered moderate according to Altman’s classification [23]. Values of k = 0.45 for TRSF and 0.48 for SSSF and ∑SF were significant (p < 0.001). In the same way, k values were moderate for boys and girls respectively: TRSF (0.45 and 0.44); SSSF (0.55 and 0.40); ∑SF (0.53 and 0.42), all values were statistically significant (p < 0.001).

**Fig 2:**
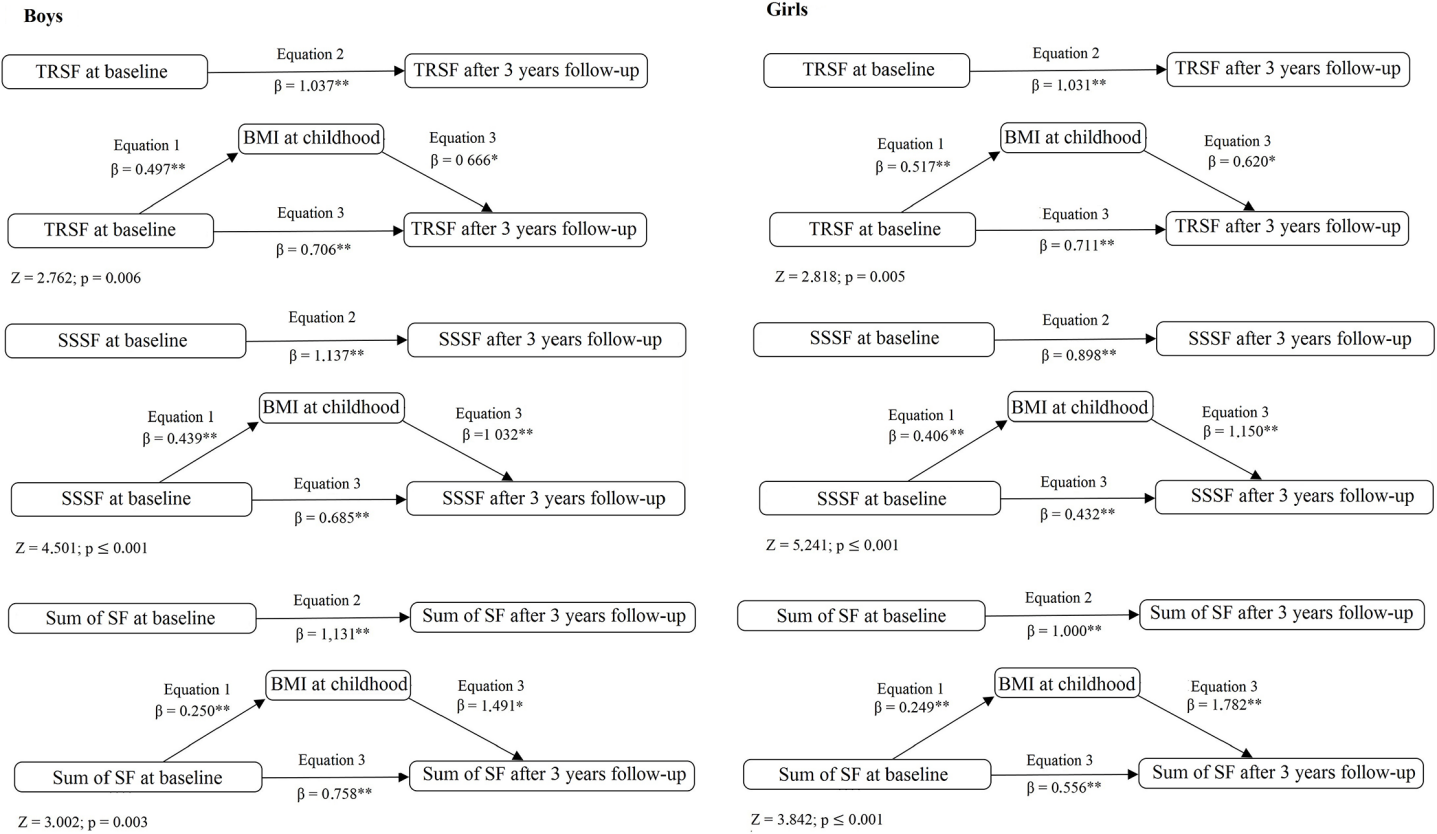
Mediation models by BMI at the age of adiposity rebound of the relationship between adiposity in childhood (baseline) and adiposity in adolescence (3 years follow-up) adjusted by chronological age and sex. Note. BMI, body mass index; TRSF, triceps skinfold; SSSF, subscapular skinfold; SF, skinfolds (TRSF + SSSF) *p < 0.05; **p ≤ 0.001.

Mediation models are described in Fig 2. In all models, BMI in childhood partially mediated the relationship between adiposity in childhood and adiposity in adolescence. In the case of ∑SF, it was observed that BMI presented mediation effects of 33% and 44% for boys and girls respectively. Concerning the Sobel test of effects, the mediation effects were higher for SSSF for boys (z = 4.501; p < 0.001) and girls (z = 5.241; p < 0.001).

## Discussion

Our main findings indicate that body adiposity indicators presented high tracking for both sexes, indicating few crossings in individual paths of the analyzed variables between childhood and adolescence. Another fact was that the relative position of subjects within groups was moderate; indicating that more than 70% of the subjects observed in the tertile of the highest adiposity in childhood remained in the same condition toward adolescence. Moreover, BMI at the age during childhood (theoretically after adiposity rebound) partially mediated the maintenance of body adiposity during the 3 years of follow-up.

We observed that body adiposity indicators increased significantly in both sexes between childhood and adolescence, with an incremental rate varying between 30% and 50%. This finding was somewhat expected, since the kinetics of skinfolds during the growth process indicate that they remain stable from around 4 to 7 years of age, then increase from this age to 12 or 13 years for boys, while for girls the increase seems to be linear up to the end of adolescence [25]. Other longitudinal studies also observed the same behavior in this age group for trunk folds as well as member folds in both sexes [12,26].

The increase in body adiposity, which was observed along the transition between childhood and adolescence in both sexes points to the importance of monitoring body adiposity in this critical period. Bearing in mind that excess accumulation of body fat is associated with several metabolic dysfunctions, such as cardiovascular diseases (i.e., hypertension, type 2 diabetes, hypercholesterolemia, hyperlipidemia, and others) [1–3]. Another issue regarding body adiposity in childhood and adolescence is the maintenance of adiposity tissue towards adulthood, given the associated behavioral factors and biology of this tissue, which can present elevated hyperplasia if stimulated [27–29], increasing, in this way, cardiovascular risk later in life [5,30,31].

Our results of self-correlation indicate high tracking for body adiposity indicators in both sexes (BMI, TRSF, SSSF, and ∑SF), regardless of chronological age. This kinetic variable indicates that individual values of body adiposity indicators present few crossings in their paths, in other words, subjects tend to maintain the individual values of adiposity on the same path during the transition between childhood and adolescence. Values of self-correlation of body adiposity indicators have been previously shown in other research, reporting elevated values (r > 0.60) with lower follow-up periods (between 3 and 8 years), while with increases in follow-up periods, a tendency to decreasing self-correlation values was observed [8,12,25,26,29,32].

Another finding of the present study was that a considerable part of sample migrated to the highest tertile of body adiposity. In this way, the maintenance of high rates of subjects in higher tertiles and the percentage change from one tertile to another of body adiposity, in general, can indicate an increase in the maintenance of the subjects into categories of risk for early development of obesity, favoring the development of several metabolic dysfunctions in childhood and adolescence, which tend to persist towards adulthood [7,33].

Our results of high self-correlation values and high maintenance rates of the subjects in higher tertiles of body adiposity can be explained through a combination of biological, environmental, and statistical factors, in an isolated or combined manner. In tracking studies, the follow-up intervals can influence continuous values of skinfolds as well as maintenance of relative positions of subjects in categorized groups. This fact occurs since the probability of a subject maintaining their position in an extreme group for a given attribute is higher when compared with those classified in intermediate groups, as the change in position in extreme groups can only occur in one direction. On the other hand, the phenomenon of regression to the mean indicates that an extreme value at one moment tends to present lower values in the posterior follow-up, in other words, the greater the interval between follow-ups, the greater the chance of the extreme values regressing to the mean of the group [34,35].

The stability of body adiposity indicators can also be influenced by possible mechanisms caused by some environmental factors, mainly dietary ingestion behavior and physical activity level over time. Even though physical activity tracking values from childhood to adolescence are moderate to low, previous studies have shown that regular practice of physical activity and sports participation are inversely related to body adiposity indicators [36–38]. In the case of dietary behavior, studies have reported that the tracking can vary from moderate to high for the consumption of high fat foods, cakes, sweets and also low ingestion of fibers, moreover, tracking of dietary patterns is associated with an increase in obesity [11,36,38]. However, these hypotheses cannot be confirmed by the present study.

Another aspect to be highlighted is that indicators of physical growth are in a transforming process during childhood and adolescence. Tracking of body size descriptors (body mass, stature and BMI) are moderate to high, which can impact on tracking of overweight and obesity measured by BMI [10–12]. Even through BMI is not a direct measure of adiposity, this indicator presents greater correlation values than subcutaneous skinfolds, which highlights the importance of stature and body size in the analysis of BMI [9]. This can be observed in the ICC for BMI in both sexes, which could be due to the magnitude of changes verified in body mass in relation to stature (42% vs. 14%).

Despite the elevated channeling of individual values of body adiposity and strong maintenance of subjects in their respective tertiles in the transition between childhood and adolescence, BMI also showed elevated channeling in the trajectory of individual values. This is an important issue because of the phenomenon of adiposity rebound, which occurs at this age (around 7 to 8 years-old). Adiposity rebound is characterized by the moment that BMI begins to increase after reaching its lowest point in early childhood, however, it is not known if changes in body adiposity are responsible for the increase in BMI [13,27].

In this way, adiposity rebound has received criticism regarding the biological basis of this theory, indicating that this phenomenon could be derived from a statistical basis, indicating a possible “horse racing effect”, which means that due to the elevated channeling of BMI during childhood and adolescence, a child with high BMI tends to persist with a high BMI during adolescence and present higher cardiovascular risk in adulthood. However, studies have related that an earlier adiposity rebound is associated with increased obesity and cardiovascular risk in adulthood even in normal ponderal children [14,39].

In this sense, our study found that the mediation effects of BMI at childhood were significant in the three variables of body adiposity in both sexes. This fact shows that BMI after adiposity rebound mediates at least part of the relationship between adiposity in childhood and adolescence, showing the importance of considering both anthropometric indicators in the tracking and prevention of obesity and cardiovascular risk later in life [14,39,40].

These results demonstrate clear practical implications. Bearing in mind the implication of obesity indicators tracking to adolescence and consequently, adulthood, early interventions in childhood should be conducted. School-based interventions with physical activity, aiming to increase physical fitness and decrease body fat and metabolic indicators have shown promising results, reducing body fat and increasing physical fitness [41,42].

Some limitations of this study should be mentioned. Firstly, our indicator of body fat is doubly indirect and can present bias, even though only one evaluator performed the measures throughout the study. Another limitation is that we did not adjust the analyzes for important confounders of obesity indicators, such as physical activity and dietary intake [43]. Moreover, we analyzed data of adiposity by tertiles in tracking analyses, which could not necessarily reflect the state of obesity, just a greater adiposity comparing to the sample. On the other hand, we presented longitudinal data, with 3 years of follow-up of 375 children/adolescents from Brazil, which is a developing country of continental size.

Thus, our study found that obesity indicators increased during 3 years in both sexes, the tracking of body fatness was high for both sexes and children tend to maintain their channel during the transition from childhood to adolescence. Moreover, BMI at childhood partially mediated the relationship between body fat in childhood and body fat in adolescence, showing in this way, a direct relationship between adiposity rebound and adiposity in childhood.

## Supporting information

S1 Dataset Dataset of the present article. (XLS)
